# Phytoliths as an indicator of early modern humans plant gathering strategies, fire fuel and site occupation intensity during the Middle Stone Age at Pinnacle Point 5-6 (south coast, South Africa)

**DOI:** 10.1371/journal.pone.0198558

**Published:** 2018-06-04

**Authors:** Irene Esteban, Curtis W. Marean, Erich C. Fisher, Panagiotis Karkanas, Dan Cabanes, Rosa M. Albert

**Affiliations:** 1 Evolutionary Studies Institute, University of the Witwatersrand, Johannesburg, South Africa; 2 School of Geosciences, University of the Witwatersrand, Johannesburg, South Africa; 3 African Centre for Coastal Palaeoscience, Nelson Mandela University, Port Elizabeth, South Africa; 4 ERAAUB. Dept. Història i Arqueologia, Universitat de Barcelona, Barcelona, Spain; 5 Institute of Human Origins, School of Human Evolution and Social Change, Arizona State University, Tempe, United States of America; 6 The Malcolm H. Wiener Laboratory for Archaeological Science, American School of Classical Studies, Athens, Greece; 7 Department of Anthropology, Rutgers, the State University of New Jersey, Biological Sciences Building, New Brunswick, New Jersey, United States of America; 8 Center for Human Evolutionary Studies. Rutgers, the State University of New Jersey, Biological Sciences Building, New Brunswick, New Jersey, United States of America; 9 ICREA, Barcelona, Spain; Max Planck Institute for the Science of Human History, GERMANY

## Abstract

The study of plant remains in archaeological sites, along with a better understanding of the use of plants by prehistoric populations, can help us shed light on changes in survival strategies of hunter-gatherers and consequent impacts on modern human cognition, social organization, and technology. The archaeological locality of Pinnacle Point (Mossel Bay, South Africa) includes a series of coastal caves, rock-shelters, and open-air sites with human occupations spanning the Acheulian through Middle Stone Age (MSA) and Later Stone Age (LSA). These sites have provided some of the earliest evidence for complex human behaviour and technology during the MSA. We used phytoliths—amorphous silica particles that are deposited in cells of plants—as a proxy for the reconstruction of past human plant foraging strategies on the south coast of South Africa during the Middle and Late Pleistocene, emphasizing the use and control of fire as well as other possible plant uses. We analysed sediment samples from the different occupation periods at the rock shelter Pinnacle Point 5–6 North (PP5-6N). We also present an overview of the taphonomic processes affecting phytolith preservation in this site that will be critical to conduct a more reliable interpretation of the original plant use in the rock shelter. Our study reports the first evidence of the intentional gathering and introduction into living areas of plants from the Restionaceae family by MSA hunter-gatherers inhabiting the south coast of South Africa. We suggest that humans inhabiting Pinnacle Point during short-term occupation events during Marine Isotope Stage (MIS) 5 built fast fires using mainly grasses with some wood from trees and/or shrubs for specific purposes, perhaps for shellfish cooking. With the onset of MIS 4 we observed a change in the plant gathering strategies towards the intentional and intensive exploitation of dry wood to improve, we hypothesise, combustion for heating silcrete. This human behaviour is associated with changes in stone tool technology, site occupation intensity and climate change.

## Introduction

The southern African sub-region provides some of the richest archaeological records for a key phase in the evolution of modern humans, dating between ~160–40 ka, when modern humans evolved, began displaying advanced behaviours, and then dispersed from Africa [1]. These advanced behaviours include the systematic exploitation of marine resources [2,3], heat-treatment of lithic raw materials [4–6], shell bead production [3,7], bone tool technology [8–10], the engraving of objects such as ochre nodules, faunal remains and ostrich eggshell [3,8,11–18], the use of pigments [2,15,19], and early microlithic technology and perhaps advanced projectile weapons [20,21].

The south coast of South Africa is located within the Greater Cape Floristic Region (GCFR) [22], a floral biodiversity centre with high levels of endemic plant species. It has been argued that the GCFR provided uniquely diverse resources that may have supported human populations throughout the Pleistocene, and especially during glacial periods when most terrestrial environments would have lowered productivity [23,24]. These include a triumvirate of a high variety of edible plants such as fruits and geophytes (plants with edible underground storage organs) [25,26], a rich marine ecosystem [27,28], and ungulate grazer populations occupying the now submerged Palaeo-Agulhas plain [29,30].

**Fig 1:**
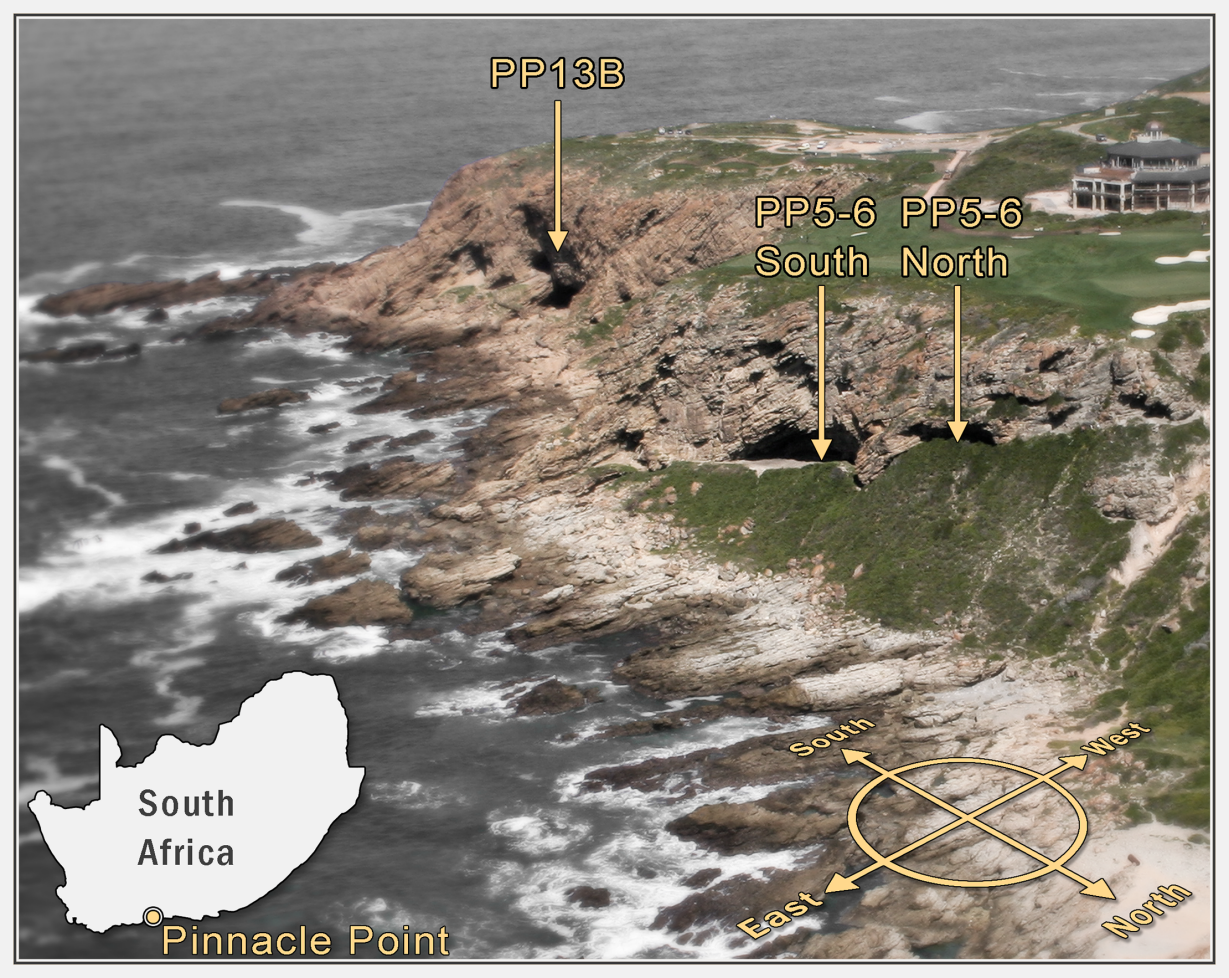
Pinnacle Point geographical location. Map of South Africa and the location of Pinnacle Point and an aerial and panoramic photograph of cave Pinnacle Point 13B and rock shelter Pinnacle Point 5-6N.

Since 2000, the South African Coast Paleoclimate, Paleoenvironment, Paleoecology, Paleoanthropology (SACP4) team has conducted multidisciplinary studies focused on the Mossel Bay region (Western Cape Province, South Africa) and specifically the Pinnacle Point caves and rock shelters on the central south coast of South Africa. Pinnacle Point (-34.207767, 22.085489) is a rocky headland on a cliffed coast on the Indian Ocean, approximately 10 km west of the Mossel Bay point (Fig 1). Although Pinnacle Point is a formal geographic location, where currently there is a large golf and holiday resort settled above the cliffs, we consider it as an area around which are concentrated a wide variety of archaeological, paleontological, and geological localities of scientific interest (Fig 1).

The archaeological work conducted at Pinnacle Point has focused on Pinnacle Point 13B (PP13B), with a discontinuous sequence from ~170–90 ka [31], that connects with the high resolution and continuous sequence of Pinnacle Point 5–6 (PP5-6) that spans ~92–49 ka [32,33]. PP5-6 includes a northern area (PP5-6N) and a southern area (PP5-6S) that we think originally connected stratigraphically, but we have yet to conduct the excavations required to determine this. PP5-6N is partially under a rock shelter and PP5-6S includes a cave. We originally separated PP5-6N into three disconnected sedimentary packets–the Northwest Remnant, the Long Section, and the Southwest Remnant. In excavations in 2017 successfully connected these three sedimentary packets and we now refer to them as PP5-6N. Together these sites make Pinnacle Point a long composite Middle Stone Age (MSA) record of human occupation spanning the early parts of Marine Isotope Stage (MIS) 6 through MIS 3. This long excavated record is rare on the South African coast.

Plants are one of the natural resources most commonly used in the daily activities of hunter-gatherer societies; as a source of food and water, as a fuel for fire, to create tools for hunting, fishing, storage and transport of food and goods, to build shelter and protection, etc. Hearth remains are a recurrent feature in hunter-gatherer archaeological sites, and this is noteworthy at Pinnacle Point where well-preserved combustion features at both PP13B and PP5-6N are present. The use and control of fire is a significant technological advance for the physical, social, and cognitive evolution of the genus *Homo* [34–37]. The importance of fire for past hunter-gatherer populations lies in its use as a tool for cooking, light, protection, heating and socialization of groups [34–38]. Understanding fire’s nature and use, as well as the study of the type of fuel used for fires, is of major importance for shedding light on its impact on early modern human gathering strategies and food and fuel preferences [39]. In South Africa, there are various MSA sites where charcoal, phytoliths, and mineralogical and geoarchaeological studies have been conducted to understand changes in wood selection, site maintenance and daily activities and past environments. At Sibudu, where hearth remains have been intensively studied, hearths associated with ochre powder production were recorded during the post-Howiesons Poort occupations [40]. At PP13B, Albert and Marean [39] interpreted the abundance of dicotyledonous leaf phytoliths during MIS 6 occupations as indicative of fires with specific properties, short-term fire activities or an unknown way of using fire in activities such it could be cooking.

We know little about the use of plants as food in the South African MSA. Charred seeds have been recovered from a few MSA South African sites, and these are Boomplaas Cave [41,42], Die Kelders Cave 1 [43], Sibudu Cave [40,44–46] and Wonderkrater [47]. Geophyte remains have been found in a variety of Holocene archaeological sites from South Africa, and some examples are Melkhoutboom [48], Scott’s Cave [49], Sehonghong [50] and Boomplaas [51,52], and De Hangen, Andriesgrond Cave and Diepkloof [53–57] in the West Coast, where evidence of consumption of *Watsonia* sp., *Moraea* sp., *Babiana* sp. and *Hesperantha* sp. (Iridaceae), and leaf sheaths of *Hypoxis* sp. (Hypoxidaceae), among other less common taxa, have been recorded. Deacon [48,58,59] proposed that the presence of geophyte remains in archaeological sites dating from the Holocene located in the Cape Floral Region indicated that people were aware of the high production of underground organs by geophytic plants and managed their productivity with fire. In Strathalan Cave B plant remains of *Watsonia* sp. were found, dating to the Terminal Pleistocene [60,61]. Despite the fact that there is one site with evidence of geophyte consumption in the MSA (Bushman Rock Shelter, see [62] various researchers have argued that geophytes would have been an important component of MSA hunter-gatherer diet [24,26,29,48,57,63–66]).

Plant remains in archaeological sites are most commonly identified as charcoal, seeds, pollen, starches, phytoliths and non-pollen palynomorphs. Here we use phytolith analysis–microscopic particles of amorphous silica formed in plant tissues–to study the use of plants during the MSA at Pinnacle Point. Phytolith analyses applied to MSA and Middle and Upper Palaeolithic sites have proved to be a powerful tool to study fuel use in fires [39,67–70], plant consumption [71] and the construction of bedding and living floors [72–74]. Due to their inorganic nature, phytoliths are resistant to most biostratinomic and post-depositional processes, including burning. Their characteristic morphologies allow for the identification of the original plant to taxonomic and anatomical level [75–81]. Notwithstanding the rich archaeological record for the South African MSA, only three sites have been studied through phytolith analysis to date and these are Sibudu Cave (KwaZulu-Natal) [69,82], Wonderkrater (Limpopo) [47] and PP13B at Pinnacle Point (southern Cape) [39].

This paper aims to investigate the evolution of plant exploitation strategies by early modern humans in the south coast of South Africa in the long composite Pinnacle Point sequence, spanning ~120,000 years. For that purpose, we use phytolith analysis at a high temporal-spatial resolution to identify changes in human behaviour and adaptation across this long time span by adding new data from PP5-6N to the previously published data from PP13B [39].

### Archaeological background

The caves and rock shelters at Pinnacle Point occur in quartzite of the Table Mountain Sandstone formation, which is overlain by calcrete formations across much of the area. All of the caves and rock shelters were eroded by ancient high sea levels. Uranium-lead dating on speleothem and thermal transfer optically stimulated luminescence (TT-OSL) on cemented marine sands provide concordant evidence that some of the caves were forming at least by ~1.1 Ma [83,84]. The peak of the MIS 11 high sea stand at ~400 ka in this area was at ~+13 msl [85] which would have washed out most of the >400 ka sediments from the caves, though some of the sites have portions that are sufficiently high to preserve older sediments.

Pinnacle Point has a long record of MSA occupation that has been excavated with high-resolution techniques including total station 3D plotting of all archaeological materials, stratigraphy, and samples, which include samples for phytolith analysis. So far, the earliest numerically dated human occupations began ~170–160 ka at PP13B and this is the only coastal occupation dated to MIS 6 on the south coast of South Africa. The record at PP13B extends to ~92 ka, at which time it is closed from further occupation by a dune [86,87]. The Pinnacle Point record continues then at PP5-6N, in a sequence that forms on top of a dune, dated ~92 ka and continues until ~49 ka [32,33].

The excavations at PP5-6N reveal a ~15 vertical meter excavated section spanning ~40 meters long horizontally. Our age estimates provided here are from a Bayesian age model calculated from 90 OSL age estimates, the identification and age of the Younger Toba Tuff via cryptotephra analysis (87), and U-Th ages of speleothem intercalated with sediments from a range of sites at PP (31, 134). The ranges mostly reflect the 1 sigma range around the oldest and youngest OSL ages rounded to a thousand years, though some estimates are more precise where other techniques (such as cryptotephra correlation to Toba) offers more precision. The sequence documents a turnover from aeolian-dominated sedimentation (YBS, ~92 ka) to roofspall-dominated sedimentation (YBSR and LBSR, ~92–75 ka), to aeolian-dominated sedimentation (ALBS to OBS2, ~74–60 ka), back to roofspall-dominated sedimentation (BAS and BBCSR, ~63–51 ka), with a final aeolian sedimentary event at ~52–49 ka [32,33]. Occupation intensities rise with the glacial MIS 4 (SADBS) [32], while at the same time there was a shift to intensive use of heat-treated silcrete [4] and the world’s earliest known microlithic technology [21]. Evidence for mollusc exploitation fluctuates through the sequence in sync with sea level and coastline changes [88].

## Materials and methods

### Materials

**Fig 2:**
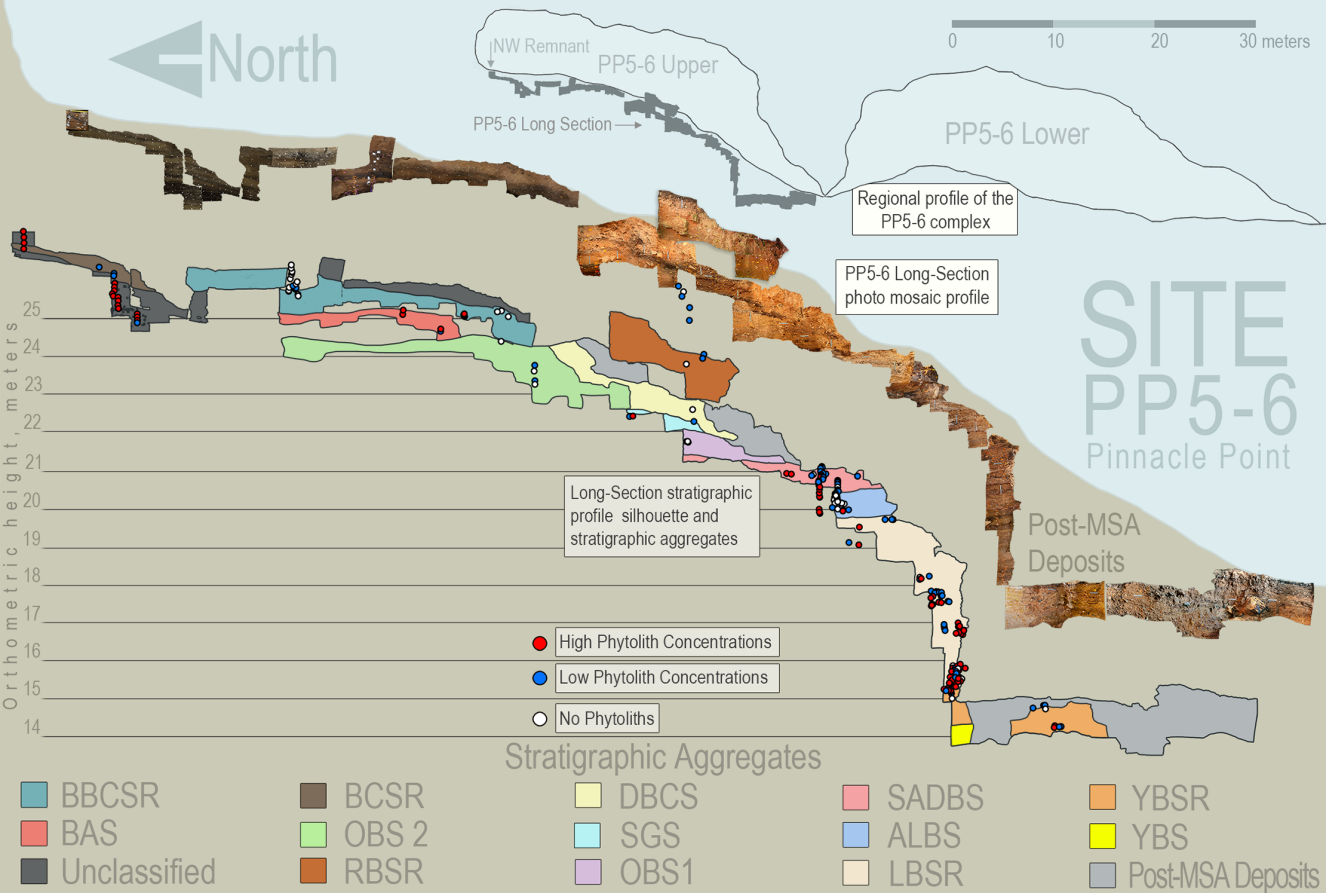
Phytolith samples location and concentration. Long Section stratigraphic silhouette showing the stratigraphic aggregates and sample location (bottom) giving distribution of phytolith presence and preservation (modified after 31, with the BBCSR and BAS sediment profiles, phytolith sample location and concentration information added). This figure is similar, but not identical, to the original image and is therefore for illustrative purposes only.

A total of 206 sediment samples were collected from PP5-6N belonging mainly to combustion features and other general anthropogenic layers. In addition, 23 control samples were collected from different geogenic layers in order to establish whether the plant phytolith remains found in archaeologically-rich layers represent anthropogenic input. Samples are stored at the Laboratory of Archaeology of the University of Barcelona (Spain), except for samples from the BBCSR and BAS Stratigraphic Aggregates (hereafter–StratAggs), which are stored at the Laboratory of Palaeobotany of the Evolutionary Studies Institute of the University of Witwatersrand (South Africa). Fig 2 shows the StratAggs of PP5-6N –representing a generally homogeneous set of sedimentary processes (modified after 32)–and the location of the 229 phytolith samples and concentration of phytoliths in samples.

**Fig 3:**
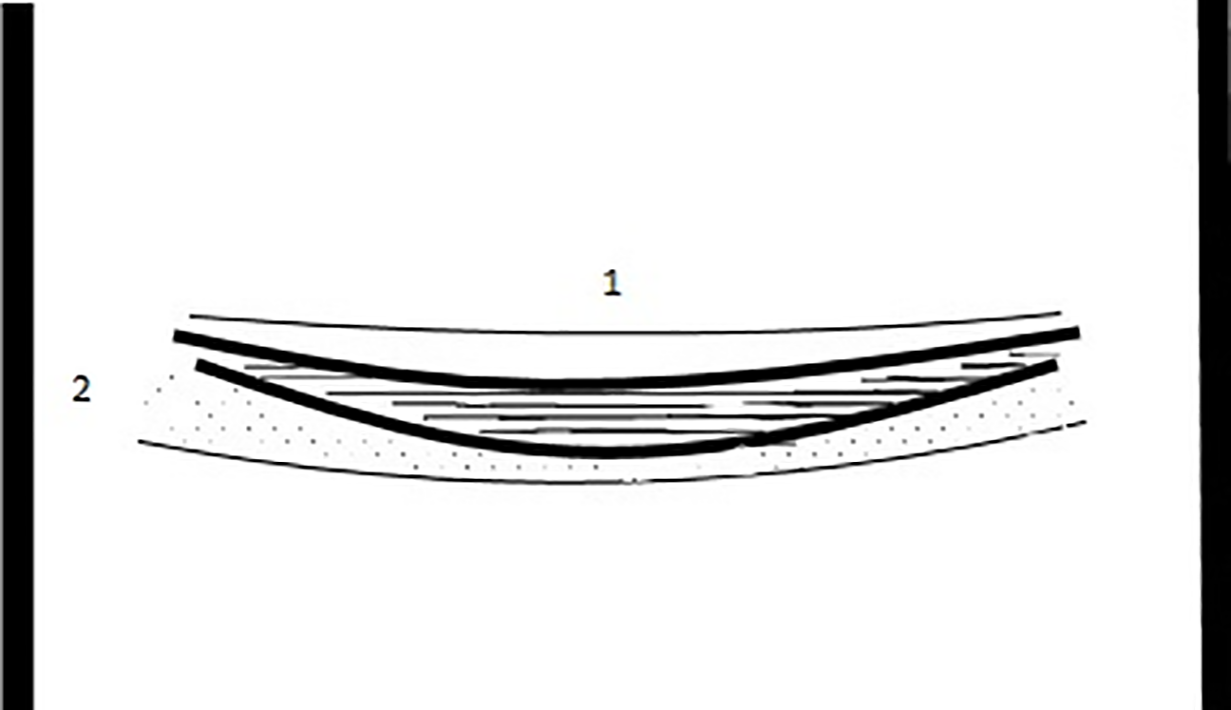
Layers of an ideal combustion feature. Schematic drawing of an ideal combustion feature showing the three combustion layers, these being the white (blank), black (horizontal lines) and red (dots) layers, and the sample location of the control samples from above (1) and outside (3) hearth.

Samples from each combustion feature were collected from three different layers within the combustion feature, which are from top to bottom the white, black and red layer. Samples from above and outside the identified hearths were also collected whenever possible as control samples. The red layer is understood as the sample from below hearths. This layer is produced by the reddening of the underlying sediments when the combustion feature is active [68,89]. Fig 3 shows the schematic drawing of an ideal combustion feature and layers. This sampling procedure was mainly possible in the YBSR and LBSR StratAggs where practically intact combustion features were preserved as the result of high and relatively constant rate of geogenic input, particularly roofspall, together with low rates of anthropogenic activity, such as trampling, that prevented their destruction [32]. Conversely, in the SADBS, complete single combustion features were not easily discernible since this StratAgg consists of a thick sequence of overlapping trampled combustion microfacies (microscopic facies) with a few isolated lenses of intact combustion features identified mostly microscopically [32].

### Sampling methods

PP5-6N has been excavated since 2006. Excavation permits were issued by Heritage Western Cape and landowner permission was granted by Pinnacle Point Estate. Excavation methods are described in Dibble et al. [90], Marean et al. [31], Bernatchez and Marean [91] Oestmo and Marean [92], and Fisher et al [93]. All sediment samples recovered for phytolith analysis were plotted directly with a total station in three dimensions. A bar-code number is given to every sample plotted. This is the sample number used for sample identification.

### Phytolith extraction

The phytolith extraction from the archaeological sediments was carried out at the Laboratory of Archaeology of the University of Barcelona (Spain) and partially at the Laboratory of Palaeobotany of the Evolutionary Studies Institute of the University of Witwatersrand (South Africa) following the fast extraction procedure of Katz et al. [94]. An initial sediment weight of between 30 and 50 mg was required. Carbonate minerals were dissolved adding 50 μl of hydrochloric acid (6 N HCl). After the bubbling ceased, 450 μl of 2.4 g/ml sodium polytungstate solution Na_6_ (H_2_W_12_O_40_)·H_2_O] was added. The tube was vortexed, sonicated and centrifuged for 5 min at 5000 rpm (MiniSpin plus, Eppendorf). The supernatant was subsequently removed to a new 0.5 ml centrifuge tube and vortexed. For examination under the optical microscope, an aliquot of 50 μl of the supernatant was placed on a microscope slide and covered with a 24 x 24 mm cover-slip. Quantification of the total phytoliths was based on 20 fields at 200x magnification whereas morphological identification of phytoliths took place at 400x magnification using an optical microscope (Olympus BX41). A minimum of 200 phytoliths were counted for the morphological analysis and when this was not possible only those samples with a minimum number of 50 phytoliths were analysed in order to obtain as much information as possible [95]. However, the error margin in the interpretation of the phytolith representation when using a minimum of 50 phytoliths is very high (40%, Albert and Weiner, 2001).

### Phytolith classification

Morphological identification of phytoliths was based on our modern reference material from the study area, from plant species [96] and modern surface soils [97]. The results were also compared to other modern phytolith reference South African collections of plants [82,98–101] and modern surface soils [99–101]. Reference collections from other African regions [102–108] and the PhytCore database (www.phytcore.org, [109]) have also been consulted. Additionally, standard literature [75–77,110] was accessed when necessary. The terminology for describing phytolith morphotypes was based on the anatomical and/or taxonomic origin of the phytoliths. When this was not possible, geometrical traits were followed. The International Code for Phytolith Nomenclature (ICPN) was also followed where possible [111].

### Statistical analysis

The non-parametric Kruskal-Wallis test, followed by a post-hoc Dunn’s pairwise comparison test with Bonferroni adjustments, were performed to determine statistically significant differences in the distribution of phytoliths representative of different plant types among the different occupation periods (StratAggs) at PP5-6N since the data were not normally distributed (Shapiro-Wilk test for normality). The significance level was set at *p* = < 0.05.

To determine the degree of preservation of the phytolith assemblages at PP5-6N we used non-parametric Spearman’s correlations to measure the strength of the association and the direction of the relationship, in association with a *p-value* computation, between the phytolith concentration (per gram of sediment) and three taphonomic indicators, i) the total number of morphotypes identified [112,113], ii) the percentage of weathered morphologies–non-recognizable phytoliths with signs of chemical dissolution and/or mechanical damaged–and iii) the percentage of fragile morphologies–which are the first ones to disappear under certain post-depositional processes–such as hair cells, stomata, papillae [114,115], tracheids, epidermal cells (articulated phytoliths) and parenchyma strands from dicotyledonous plants (hereafter–dicots) (e.g, [76,77]) for each StratAgg.

All analyses were computed using JMP-SAS 13.2.1 software.

### Mineralogical analysis

Fourier Transform Infrared Spectroscopy (FT-IR) was used to identify the bulk mineral components of the archaeological sediments in order to, 1) understand the conditions that may have affected the state of preservation of phytoliths and, 2) further shed light on fire use and site occupation patterns. Infrared spectra were obtained using KBr pellets at 4 cm-^1^ resolution with a Nicolet iS5 spectrometer. In order to assess the origin of the calcite, we have applied the infrared grinding curve method developed by Regev et al. [116] based on the measurement of the ratio of *v*_*2*_/*v*_*4*_ heights (1420 cm^-1^ and 713 cm^-1^, respectively) normalized to a *v*_*3*_ height (874 cm^-1^). Clays exposed to high temperatures were identified using changes in the specific absorptions bands of the clay spectrum [117].

## Results

Table 1 lists the sixty-three samples with a minimum number of recognizable phytolith morphotypes (>50), together with their stratigraphic location and description, and the main phytolith and mineralogical results. The description of samples with insufficient number of identifiable phytoliths is given in Supplementary Material (S1 Table). The phytolith morphotypes identified, taxonomic association and its frequencies in samples from the different StratAggs are listed in S2 Table. Sixty-two phytolith morphotypes were identified [see phytolith morphotype descriptions in Esteban [118], which were later grouped by plant types and plant parts into twelve general categories: grasses (Poaceae), restios (Restionaceae), sedges (Cyperaceae), palms (Arecaceae), leaves, wood/bark and fruits of dicots, spheroids (non-decorated margins), stomata, elongates with and without decorated margins, and irregular and indeterminate morphologies. Note that the term dicot was used for all non-monocotyledonous (hereafter—monocots) angiosperms because based on phytoliths it is difficult to distinguish between early-diverging angiosperms and eudicots. Despite non–decorated spheroid morphologies have been typically associated to the wood/bark of dicot and other non-flowering plants (i.e. Gymnosperms) (e.g., [95,109,119,120]), they were grouped separately as they constitute an important component also in restios [96,97].

**Table 1:** Main phytolith and FT-IR results giving sample description and provenance.

Sample Number	Stratigraphic Aggregate		Sample Type	# Phytoliths counted	Phytoliths /g sed	% WM	% Diatoms	% Sponge spicule	FT-IR
**418155**	**BBCSR**	**Black and Brown Coarse Sand and Roofspall** dating ~63–51 ka This StratAgg shows coarse sand and the presence of roofspall. This StratAgg is also characterized by the alternation of intense occupation layers with layers showing lower occupancy.	White layer	112	135,100	46.43	0	0	Cal, Cl (b), Qtz, Dah
**602416**	**BBCSR Inside Dripline**		Black layer	109	350,861	25.34	1.80	0	Qtz, Cl (nb), Nit
**602417**	**BBCSR_ Inside Dripline**		Black layer	56	299,739	47.66	0	0	Qtz, some Cl (nb), Nit
**602418**	**BBCSR_ Inside Dripline**		Black layer	165	1,054,773	13.16	2.37	0	Qtz, Cl (nb), Nit
**602419**	**BBCSR_ Inside Dripline**		Black layer	133	465,095	18.40	0.75	1.48	Qtz, Cl (nb), Arg, Nit
**630356**	**BBCSR_ Inside Dripline**		Black layer	78	1,641,401	63.21	0	1.27	Cal, Qtz, Cl (b?), Dah
**630357**	**BBCSR_ Inside Dripline**		Black layer	172	887,953	22.17	0	0	Cal, Qtz, Cl, Dah
**602403**	**BAS**	**Black Ashy Sand** dating ~63–60 ka Formerly unrecognized as it was not intersected until it was excavated further to the north of the Long Section in 2016. This StratAgg is finely bedded to laminated with more lightly grey and dark brown and black ashy layers. It has an overall thickness of half a meter or more in some places. It displays an intense occupation with dense finds and combustion features.	Black layer	190	2,139,398	22.45	0.52	1.55	Cl (nb), Qzt, Nit
**602404**	**BAS**		Black layer	94	691,789	28.46	0	1.06	Qtz, Cl (nb), Nit
**602405**	**BAS**		Black layer	142	2,210,979	14.97	5.33	1.39	-
**602406**	**BAS**		Black layer	203	2,084,237	6.45	3.33	1.46	Cl (b), Qtz, Dah, few Cal, Nit
**602407**	**BAS**		Black layer	61	440,357	20.78	0	0	Qtz, few Cl
**602408**	**BAS**		Black layer	207	1,176,495	12.66	1.43	0.96	Cl (nb?), Qtz, Cal, Dah
**602409**	**BAS**		Black layer	204	3,121,508	7.27	1.45	1.92	Cl (nb), Qtz, Cal (spar), Dah
**602410**	**BAS**		Black layer	219	3,396,143	10.98	3.52	2.67	Qtz, Cl (b?), Cal, Dah
**602411**	**BAS**		Black layer	181	1,084,625	9.50	4.74	3.72	Qtz, Cl (nb), few Cal
**602412**	**BAS**		Black layer	184	1,280,862	8.91	6.60	5.15	Cl, Qtz, some Ca
**602413**	**BAS**		Black layer	124	1,058,801	8.82	7.46	6.77	Arg transforming to Cal, Qtz, Cl (nb), some Dah
**602414**	**BAS**		Black layer	120	1,223,935	22.67	3.33	3.33	Cal, Qtz, Cl (nb), Dah?
**630351**	**BAS**		Black layer	73	626,893	51.97	0	0	Cal, Qtz, Cl (b), Dah
**630364**	**SGS**	**Shelly Grey Sand** dating ~67–62 ka This StratAgg is composed of varying light red to dark brown aeolian sand with a significant component of grey ash and shell, interstratified with layers of orange to light brown Aeolian sand with few finds.	Black layer	62	106,429	17.74		17.74	Cal, Cl (b), Qtz, some Dag
**630334**	**SADBS Inside Dripline**	**Shelly Ashy Brown Sand** dating to ~73–68 ka This is a crudely bedded grey sandy sediment with significant input of ash and moderate amounts of centimetric roofspall. It displays very high occupation intensity with significant evidence for combustion and trampling. A shift in cultural material to microlithic technology and heavy emphasis on heat-treated silcrete is documented.	Black layer	87	294,124	20.91	1.14	5.43	Arg, Cal, Qtz, Cl, Dah
**630338**	**SADBS_ Inside Dripline**		Black layer	141		16.07	0	0	Cal, Qtz, Cl (nb)
**630339**	**SADBS_ Inside Dripline**		Black layer	157	241,078	14.21	1.26	0	Cal, Qtz, Cl (nb), Nit
**630340**	**SADBS_ Inside Dripline**		Black layer	111	407,978	11.90	0.89	0.89	Cal, Qtz, Cl (nb), Nit
**630341**	**SADBS_ Inside Dripline**		Black layer	168	556,334	10.64	0	0	Cal, Qtz, Cl (b?), Nit
**630342**	**SADBS_ Inside Dripline**		Black layer	71	806,469	26.04	0	0	Qtz, Cal, Nit, Cl (nb)
**630343**	**SADBS_ Inside Dripline**		Black layer	225	434,586	9.64	0.88	0.44	Ca, Qtz, Nit, Cl
**630344**	**SADBS_ Inside Dripline**		Black layer	137	555,545	10.46	0	0.72	Arg transforming to Cal, Qtz, some Cl, Dah
**630345**	**SADBS_ Inside Dripline**		Black layer	143	621,240	8.92	0	0	Arg transforming to Cal, Qtz, Cl (b), Dah
**630346**	**SADBS_ Inside Dripline**		Black layer	153	274,942	13.53	0	0	Cal, Qtz, Cl, Nit
**162466**	**SADBS**		White layer	214	300,000	30.37	0	0	Cal, Qtz, Cl (b), Dah
**162467**	**SADBS**		Black layer	93	69,000	24.73	0	0	Cal, Qtz, Cl (b), few Dah
**46682**	**SADBS**		Black layer	75	67,000	37.33	0	0	Cal, Qtz, Cl (nb), some Dah
**356487**	**SADBS**		White layer	59	75,100	72.88	0	0	Cal, Qtz, Cl (b?), few Dah
**356491**	**SADBS**		Grey colour	86	78,900	27.91	0	0	Cal, Qtz, Cl (b), some Dah
**630333**	**ALBS_ Inside Dripline**	**Aeolian Light Brown Sand** dating to ~74 ka This is an aeolian sand dune with shell rich layers. The contact of the LBSR and ALBS represents the transition from MIS5 to 4, showing a shift from roofspall dominated sediments (LBSR) to aeolian sand dominated sediments. The dominance of aeolian sand is interpreted to reflect the coast being further away than during the LBSR.	Black layer	131	262,272	3.68	0	5.76	Arg transforming to Cal, Qtz, Cl (nb) some Dah
**630335**	**ALBS_ Inside Dripline**		Black layer	69	348,274	37.84	0	2.82	Cal, Qtz, Cl (b?), Nit
**630336**	**ALBS_ Inside Dripline**		Black layer	71	345,212	16.47	0	13.41	Arg transforming to Cal, Qtz, Cl (nb)
**357374**	**ALBS**		Grey colour	60	58,200	21.67	0	2.08	Cal, Qtz, Cl (nb)
**357383**	**ALBS**		Grey colour	202	284,100	28.71	3.36	34.25	Arg transforming to Cal, Qtz, Cl (nb)
**357380**	**ALBS**		Grey colour	202	230,300	21.78	0	2.47	Cal, Qtz, Cl (nb)
**162483**	**ALBS**		Red layer	153	123,700	34.64	1.96	6.54	Cal, Qtz, Cl (nb)
**162481**	**ALBS**		Black layer	216	314,600	18.98	0	9.33	Cal, Qtz, Cl (nb)
**630329**	**LBSR**	**Light Brown Sand and Roofspall** dating to ~89–75 ka Roofspall dominates the sediments in this StratAgg. Layers of occupation interspersed with layers of low to non-occupation alternates along this StratAgg. Layers with occupations have significant amounts of shells, some of them representing shell middens. Karkanas et al. (2015) interpreted the roofspall dominance to be the result of sea-spray and mist initiated cliff weathering when the coast is close to the site.	Black layer	52	135,993	44.09	1.89	3.70	Qtz, Cl (nb) Cal, Nit
**630331**	**LBSR**		Black layer	78	178,490	14.29	0	0	Cal, Qtz, Cl (nb), Dah
**162494**	**LBSR**		Outside Hearth	73	87,600	28.77	0	3.7	Qtz, Cl (nb), few Cal, few Arg, some Dah
**162493**	**LBSR**		Red layer	112	107,000	25.00	0	8.7	Qtz, Cl (nb), Cal, few Arg
**162492**	**LBSR**		Black layer	165	239,600	47.27	1.14	7.45	Arg transforming into Cal, Qtz, Cl (nb), few Dah
**356476**	**LBSR**		Black layer	112	117,400	10.71	0	5.66	Cal, Cl (nb), Qtz, Dah
**162558**	**LBSR**		Black layer	126	108,800	36.51	0	3.61	Cl (nb), Qtz, Cal, few Dah?
**162557**	**LBSR**		Red layer	149	320,100	33.56	0	1	Cal, Qtz, Cl (nb), few Dah
**162549**	**LBSR**		Red layer	161	416,000	71.43	0	8	Cl (nb), Qtz, Cal
**162550**	**LBSR**		Black layer	183	355,500	30.05	0	3.76	Cal, Cl (nb), Qtz
**162548**	**LBSR**		Above Hearth	50	122,600	32.00	0	0	Qtz, Cl (nb), Cal
**356475**	**LBSR**		White layer	278	1,117,500	76.98	0	0	Qtz, Cl (nb), few Cal
**356474**	**LBSR**		Black layer	245	392,700	24.90	1.6	2.13	Cl (nb), Qtz, Cal, few Dah
**357368**	**LBSR**		Black layer	199	833,200	18.22	0	1,97	Cal, Cl (nb), Qtz, Dah
**357369**	**LBSR**		Black layer	214	404,900	8.88	0	1.02	Cal, Cl (nb), Qtz, Dah
**357370**	**LBSR**		Black layer	225	855,350	6.22	0	0.94	Cal, few Cl (nb), few Qtz, Dah
**356470**	**LBSR**		White layer	97	161,100	22.68	0	2.60	Cal, Qtz, Cl (nb), few Dah
**356471**	**LBSR**		Black layer	220	354,000	20.91	0	0	Cal, Cl (nb), Qtz, Dah
**357363**	**LBSR**		White layer	206	3,780,500	58.25	0	2.27	Cal, Cl (nb), Qtz, Dah
**357362**	**LBSR**		Black layer	170	887,900	20.59	1.46	2.88	Cl (nb), Qtz, Cal, Dah
**357364**	**LBSR**		Black layer	132	310,800	13.64	0	7.32	Cal, Cl (nb), Qtz, Dah
**357365**	**LBSR**		Black layer	64	362,700	23.44	2	7.55	Qtz, Cl (nb), some Cal, few Dah
**357366**	**LBSR**		Black layer	138	185,600	11.59	0	1.61	Cal, Qtz, Cl (nb), Dah
**356454**	**LBSR**		Black layer	185	558,700	19.46	3.87	0	Cl (nb), Qtz
**356453**	**LBSR**		Black layer	314	866,200	6.69	3.93	1.35	Cl (nb), Qtz
**356455**	**LBSR**		Black layer	227	372,500	5.73	0	0.47	Cl (nb), Qtz, some Cal, Dah
**162778**	**LBSR**		Black layer	219	1,237,700	67.58	1.39	2.74	Qtz, Cl (nb), few Cal
**356464**	**LBSR**		Black layer	179	364,500	22.91	0	0	Cal, Cl (nb), Qtz, few Dah
**356469**	**LBSR**		Black layer	136	220,400	17.65	0	0	Qtz, Cl (nb), few Cal, some Dah
**356462**	**LBSR**		Black layer	119	135,400	5.88	0	2.61	Cl (nb), Qtz, some Dah
**356456**	**LBSR**		Black layer	90	225,100	13.33	0	3.7	Cl (nb), Qtz, few Dah
**162781**	**LBSR**		Black layer	379	1,185,500	11.08	0.59	0.59	Cl (nb), Qtz, Cal, some Dah
**356457**	**LBSR**		Black layer	212	771,000	7.55	0	3.92	Cl (nb), Qtz few Cal, Dah
**356458**	**LBSR**		Black layer	180	539,800	5.56	0.58	3.41	Cl (nb), Qtz, few Cal, Dah
**356459**	**LBSR**		Black layer	230	491,300	10	0.48	6.33	Cal, Cl (nb), Qtz, Dah
**162782**	**LBSR**		Black layer	244	255,300	29.92	0	2.84	Cal, few Cl (nb) and Qtz, Dah
**356463**	**LBSR**		White layer	77	155,100	68.83	0	0	Cal, few Qtz and Cl (b), Dah
**356460**	**LBSR**		Black layer	187	216,600	4.28	0	4.79	Cal, Cl (nb), Qtz, few Dah
**356461**	**LBSR**		Black layer	128	174,200	19.53	0	11.97	Cal, Cl (nb), Qtz, some Dah
**162717**	**LBSR**		Red layer	100	75,100	35.00	0	7.14	Cal, Cl (nb), Qtz, some Dah
**162728**	**LBSR**		Black layer	260	212,500	28.85	0	2.63	Cal, Qtz, Cl (nb), Dah
**162749**	**LBSR**		White layer	142	141,900	27.46	0	9.65	Arg transforming into Cal, Qtz, Cl (b?), some Dah
**162750**	**LBSR**		Black layer	91	117,100	42.86	0	7.14	Arg, Qtz, Cl (nb), some Cal, some Dah
**162783**	**YBSR**	**Yellow Brown Sand and Roofspall** dating to ~92–84 ka This StratAgg characterized by a very yellow to light brown coarse-grained fault breccia-dominated sediment. Roofspall of centimetric and decimetric size is abundant, sometimes even roofspall-supported. Shellfish is present in the combustion features but these are significantly less abundant than in the following StratAgg. Combustion features tend to be small to ephemeral, comprising lenses of red sediment.	Red layer	110	115,700	4.55	0	1.87	Qtz, Cl (nb)
**356479**	**YBSR**		White layer	126	935,700	15.87	0	0.93	Cal, Qtz, Cl (nb), few Dah
**388612**	**YBSR**		White layer	62	1,165,700	30.51	0	0	Qtz, Cal, Cl (nb)
**388613**	**YBSR**		Red layer	135	289,400	64.44	0	0	Qtz, Cl (nb), Cal
**388614**	**YBSR**		Grey colour	122	350,900	31.15	0	0	Cal, Qtz, Cl (b), Dah
**388615**	**YBSR**		Black layer	228	482,500	76.75	0	3.64	Qtz, Cal, Cl (nb)
**388588**	**YBSR**		Black layer	109	210,600	57.80	0	8	Cal, Cl (nb), Qtz
**356478**	**YBSR**		Black layer	111	106,000	20.72	0	3.30	Qtz, Cl (nb), few calcite
**162710**	**YBSR**		Black layer	97	117,700	21.65	0	0	Qtz, Cl (nb)
**356414**	**YBSR**		Black layer	105	143,400	9.01	0.98	5.61	Arg transforming into Cal, Qtz, Cl (nb)
**356417**	**YBSR**		Red layer	111	133,900	22.58	0	2.04	Qtz, Cl (nb), Cal

List of the sixty-three samples with sufficient number of recognizable phytoliths to be interpreted, together with their stratigraphic location and description, and the main phytolith and mineralogical results: total number of phytoliths morphologically identified, relative number of phytoliths per gram of sediment (/g sed), percentage of weathered morphologies, diatoms and sponge spicules and FTIR results. WM = weathered morphologies. Arg, aragonite. Cal, calcite. Cl, clay (b = burned), (nb = not burned), (b? = probably burned since clay absorption peaks locate in a midpoint between burned and unburned clay). Dah, dahllite. Nit, nitrate salts. Qtz, quartz.

### Mineralogy

FT-IR analyses indicated that samples were composed mainly of clay, quartz, calcite, aragonite and dahllite (carbonated hydroxyapatite) in different proportions (Table 1 and S1 Table). Nitrate salts were detected in some of the samples (Table 1 and S1 Table). The presence of nitrate minerals, identified in spectra through the sharp peak at 1,384^−1^, might come from the precipitation due to the evaporation of groundwater at the sediment surface [121]. The main differences in the mineralogical composition were related to the stratigraphic location of samples as well as to the sample types (sediment colours and hearth facies). In the lower StratAggs (YBSR and LBSR), clay, quartz and calcite were the main minerals identified, whereas dahllite and aragonite were barely present, as opposed to ALBS and SADBS. In the ALBS, quartz and clay predominated and calcite was mostly present in the white layers. Traces of dahllite and aragonite were also identified in some samples but absorption peaks were low. Samples from the SADBS (both at the and inside the dripline) differed from other StratAggs by showing the highest absorption peaks of calcite and aragonite. Dahllite was also detected in all the samples from this StratAgg (S1 Table). The origin of aragonite in archaeological sites usually derives from land snails or marine molluscs [121], such as mussels. Nonetheless, aragonite can also be formed from aqueous solution at high-temperatures [122]. Fragments of seashells, recovered from those sediment samples where aragonite was detected, were also analysed through FT-IR in order to assess the origin of the aragonite. The majority of the seashells analysed preserved aragonite, suggesting that the most probable source of aragonite in the sediment samples is marine molluscs. Finally, in samples at the dripline area of the uppermost StratAggs (OBS2, SGS, OBS1, DBCS, BBCSR, RBSR), quartz and clay dominated the mineral component and calcite was absent. Only four samples from BBCSR (418152, 418153, 418154, 418155) showed calcite as the main mineral component, absorption peaks characteristic of burned clay (following Berna *et al*., 2007) and the presence of dahllite. The samples analysed from inside the dripline at BBCSR and BAS present similar mineralogical components to the samples at the dripline, with quartz and clay being the most abundant minerals and calcite absent in most of the samples presenting low absorption peaks (Table 1 and S1 Table). Aragonite and aragonite transforming into calcite was also identified in a few samples from inside the dripline area (S1 Table).

### Phytolith concentration

Phytoliths were detected along all the samples of the PP5-6N sequence but with different concentrations (Table 1 and Fig 2). The 23 control samples collected from geogenic/sand dune layers had few or no phytoliths independently of their provenance in the sequence, and none of them reached the minimum number of phytoliths necessary to conduct a reliable interpretation of the data (S1 Table). All the samples containing enough phytoliths for a reliable morphological interpretation belonged to combustion features. Phytolith concentrations varied from the lowermost StratAggs (YBSR, LBSR, ALBS and SADBS), with LBSR and YBSR showing the highest concentration, to the uppermost StratAggs at the dripline area (OBS2 and OBS1, SGS, DBCS, BBCSR, RBSR), which were sterile with the only exception of sample 418155 from BBCSR (Table 1 and S1 Table). A new area excavated in 2017 inside the dripline at the uppermost StratAggs (BAS and BBCSR) showed the presence of phytoliths in high concentrations. Thus, the PP5-6N phytolith sequence crosses the inter-glacial to glacial transition of MIS 5 to MIS 4 where there are important documented changes in stone tools and site occupation [4,21,32], and the MIS4 to MIS3 transition that is at this time less well-known in southern Africa, though very well preserved at PP5-6N and the subject of future studies and publication.

### Phytolith preservation

Phytoliths with signs of weathering (weathered morphologies) were identified in all the samples in moderate frequencies with some exceptions as described below (Table 1). Because phytoliths mostly come from combustion features the presence of these weathered morphologies might be partly related to the effect of fire [114]. Moreover, fragile morphologies were also preserved in most of the samples (Table 2). Strikingly, samples 418155 (55%) from the BBCSR, and 162778 (22.5%) and 356463 (20.8%) from the LBSR presented high frequencies of weathered morphologies, what might be indicative of some sort of post-depositional processes affecting the phytolith assemblage, along with fragile morphologies, what might be indicative of good preservation conditions. The exceptions were samples 356487, 356475 and 162750 from the LBSR, which did not have fragile morphologies. The latter come from an area that was affected by water processes and some of the combustion features show signs of decalcification [32]. Samples from the ALBS and SADBS had low frequencies of weathered morphologies, with the exception of a white layer (356487) from SADBS, with 72.88% of weathered morphologies. In this sample the only phytoliths morphologically identified corresponded to non-fragile morphologies such as elongates, parallelepiped blockys, irregular morphologies and grass silica short cell (GSSC) rondels which are resistant morphotypes [115]. Restio phytoliths were also present, which are also suspected to be resistant morphologies [96,97].

**Table 2:** Phytolith taphonomic indicators.

	% Weathered morphologies		% fragile morphologies		Number of morphotypes	
	R	*p-value*	R	*p-value*	R	*p-value*
BBCSR	-0.1786	0.7017	-0.1482	0.7511	0.6307	0.1289
BAS	-0.4341	0.1383	0.2912	0.3344	0.5215	0.0676
OBS2	-	-	-	-	-	-
SGS	-	-	-	-	-	-
SADBS	0.5	0.391	-0.1026	0.8696	-0.1539	0.8048
SADBS_Ins_Dripline	-0.3697	0.2931	0.3091	0.3848	0.2683	0.4536
ALBS	0.1	0.8729	0	1	1	<0.0001
ALBS_Ins_Dripline	1	<0.0001	-0.5	0.6667	-0.5	0.6667
LBSR	-0.2397	0.1215	-0.0454	0.7696	0.0486	0.7542
YBSR	-0.5182	0.1025	0.1009	0.7678	0.3257	0.3284

Spearman’s correlation coefficients (R) and the *p-value* computation of the phytolith concentration per gram of sediment against, a) percentage of weathered morphologies; b) percentage of fragile morphologies; and c) number of phytolith morphotypes.

We used Spearman’s correlation coefficients analysis to measure the strength of the association between the phytolith concentration and three taphonomic indicators (% weathered morphotypes, % delicate morphologies and number of morphotypes identified), so as to investigate the degree of preservation of the phytolith assemblages at PP5-6N. No significant *p-values* were recorded for most of the measurements implying that there is inconclusive evidence about the significance of the association between the variables (Table 2). In other words, we cannot conclude that the correlation is different from 0. Correlation coefficients and *p-values* for ALBS and ALBS inside the dripline are suspected of error due to the small sample size.

### Combustion features: Lateral and vertical variation

Black layers had the highest phytolith concentration, followed by white layers (Table 1). Conversely, red layers had the lowest phytolith concentration (Table 1). We expect this since red layers represent heated zones below hearths. Samples from above and outside hearths showed the lowest phytolith concentration.

**Fig 4:**
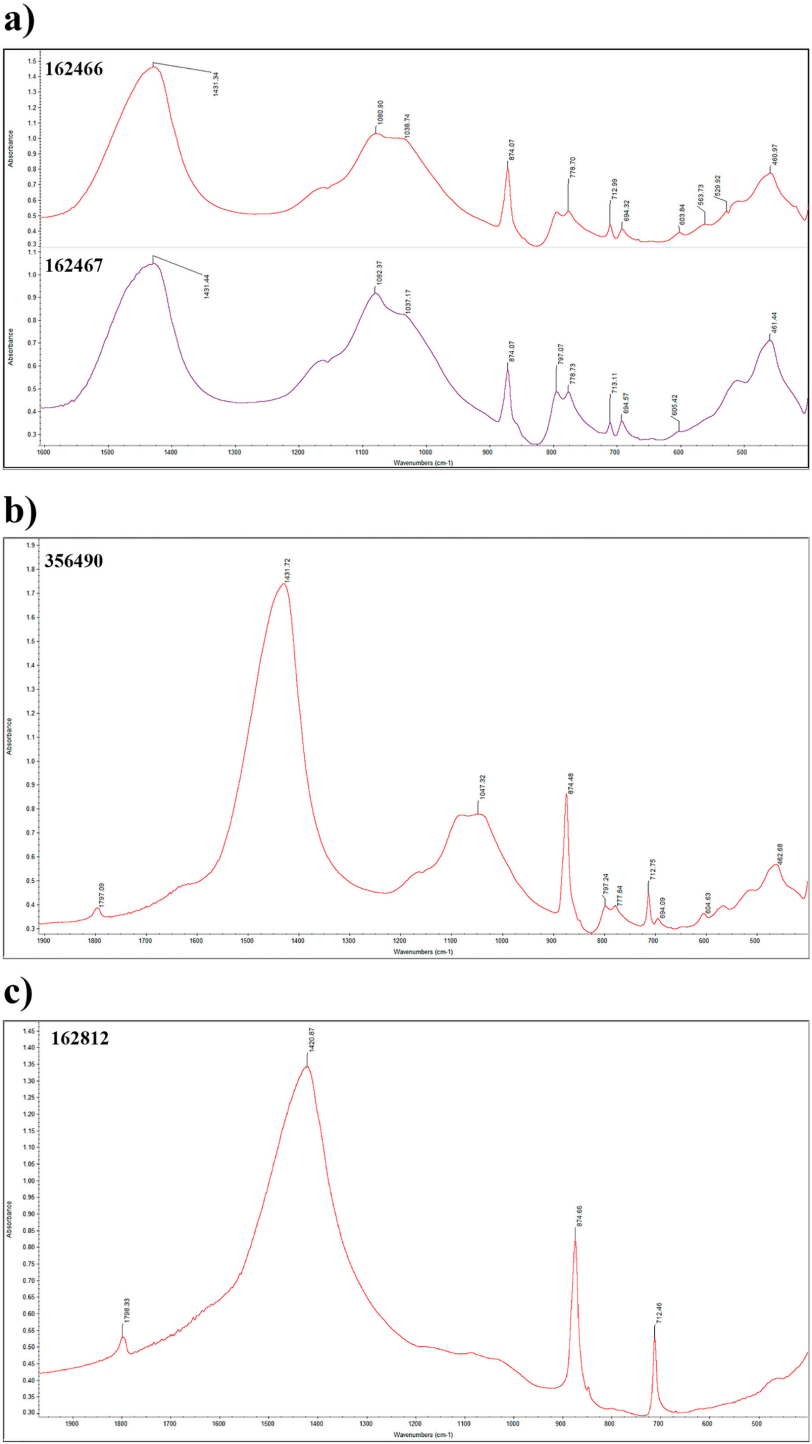
Sample mineral composition. Representative FTIR spectra of sediment samples from different StratAggs and sample types (hearth facies). a) white layer (162466) showing clay absorption peak at 1038 cm^-1^ characteristic of burned clay; b) white layer showing clay absorption peak at 1047 cm^-1^ characteristic of clay exposed to high temperatures; c) white layer showing three calcite absorption peaks at 1420, 874 and 712 cm^-1^.

Calcite was the dominant mineral component in the white layers, clay and calcite in the black layers, and samples from the red layers and above hearths contained little or no calcite, being mostly quartz and clay (Table 1 and S1 Table). FT-IR also showed traces of burned clay in most of the samples from white and black layers (Fig 4A and Table 1), and some of them at high temperatures (Fig 4B and Table 1) (following Berna *et al*., 2007). Samples from white and black layers also showed high absorption peaks of calcite (Fig 4C) which derives from wood-ash (following Regev *et al*., 2010). Wood-ash calcite was an important mineral component only in two out of the nine samples analysed from outside hearths. Phytoliths were found in low concentrations in these two mentioned samples (S1 Table). Similarly, some samples from the red layers contained calcite as the main mineral component. The presence of high calcite concentration in some of the samples from outside hearths and from the red layers might be explained by the reworking of the sediments with the black and white layers.

Samples from white layers had the highest frequencies of irregular morphotypes (phytoliths that cannot be geometrically or taxonomically/anatomically described) and the lowest frequencies of grass phytoliths (S2 Table). Samples from black layers had high frequencies of restio phytoliths in comparison with other hearth facies.

### The stratigraphic and temporal variation in phytolith morphological distribution along PP5-6N

**Fig 5:**
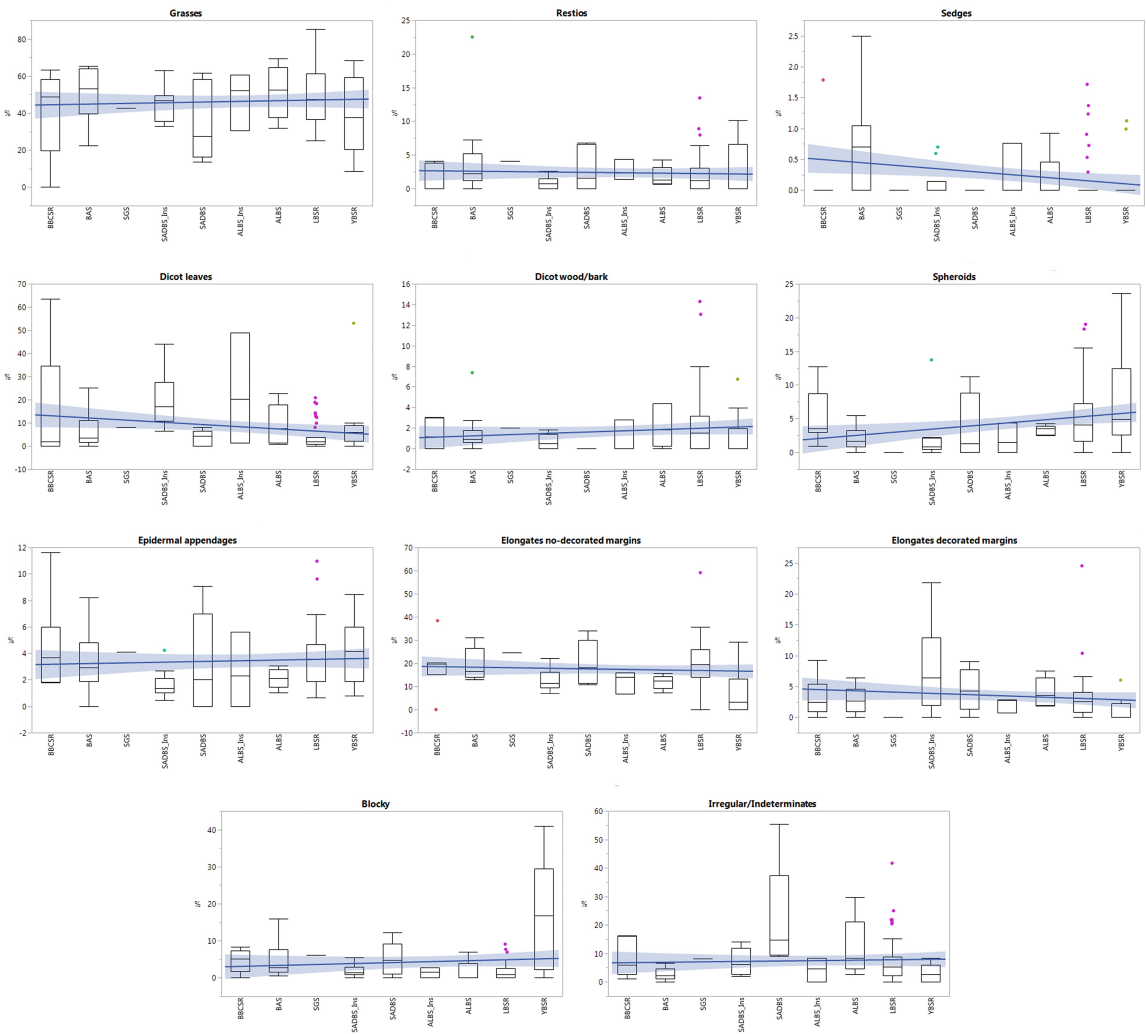
Plant type and plant parts distribution along PP5-6N. Box-plots showing the plant types and plant parts identified as significantly different among the different StratAggs at PP5-6N. The median (mid-line), standard error ± (box), standard deviation (whiskers), outliers extended beyond the whiskers, and the trend line (or line of best fit) showing the confidence region for the fitted line are given for each of the plant groups.

Here we describe the distribution of phytolith morphologies in respect to their stratigraphic location along the PP5-6N sequence. Fig 4 shows box-plots presenting the phytolith distribution grouped by plant types in each StratAgg. Spheroid echinates are excluded from Fig 5 because they were identified in very low frequencies (S2 Table). Spheroid echinates are known to come from different plant families but only two occur in the GCFR and these are Strelitziaceae and Orchidaceae. Although Arecaceae plants produce the highest numbers of spheroid echinate phytoliths in comparison to the other subfamilies, this plant family does not occur in our study area. However, there is no reference collection on these plant families from South Africa.

**Fig 6:**
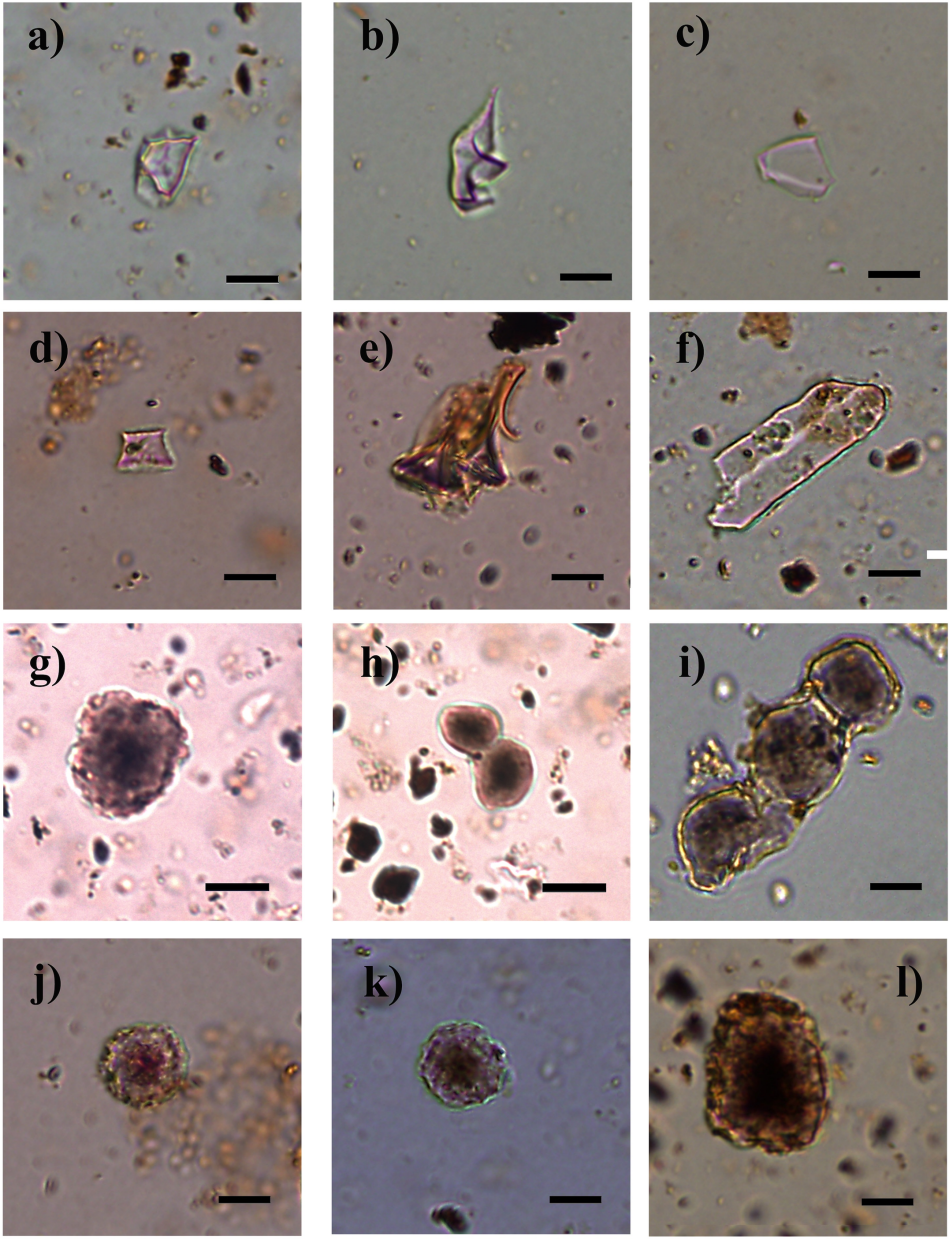
Common phytolith morphotypes. Microphotographs of common phytolith morphotypes identified in samples from different StratAggs of PP5-6N. a-c) irregular morphologies from samples 162467, 162548 and 46682 from SADBS; d-f) grass silica short cells (GSSCs): d- GSSC rondel from sample 162749 from LBSR, e- GSSC rondel tall from sample 162781 from LBSR and f- GSSC oblong tabular sinuate from sample 356455 from LBSR; g-l) restio phytoliths: g,h- sample 602414 from BAS, i- sample 356490 from SADBS, j,k- samples 162782 and 157209 from LBSR, l- sample 388612 from YBSR. Pictures taken at 400x. Scale bar represents 10 mm.

**Fig 7:**
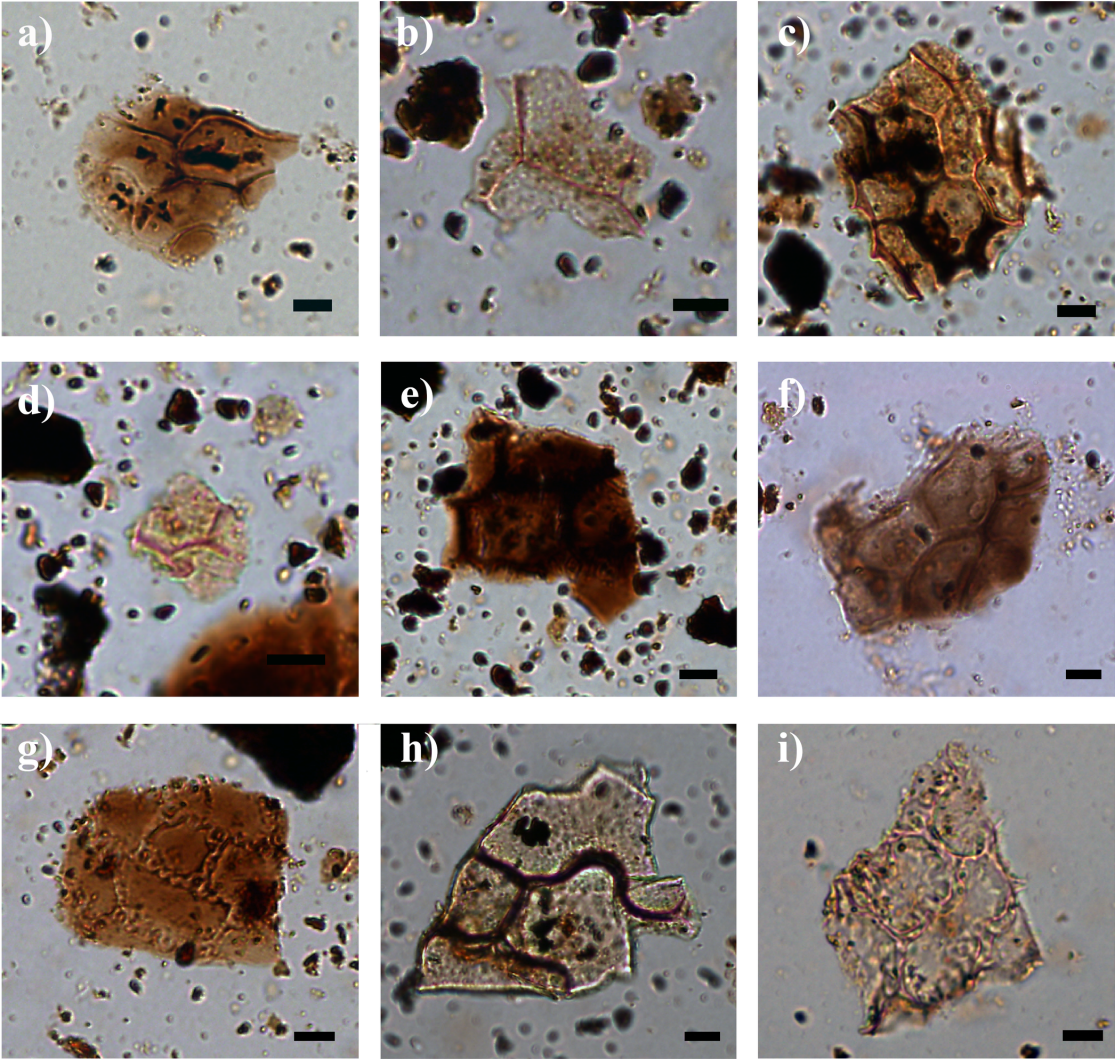
Dicot leaf phytoliths. Microphotographs of articulated phytoliths from dicotyledonous leaves showing different outlines of the cell walls from different StratAggs of PP5-6N. a) indeterminate outlines from ALBS (357374); b) indeterminate outlines from LBSR (357366); c) polyhedral outlines from LBSR (357368); d) indeterminate outlines from LBSR (357368); e) showing polyhedral outlines from LBSR (356474); f) polyhedral outlines from LBSR (162782); g) sinuate outlines from LBSR (357365); h) sinuate outlines from LBSR (162778); i) sinuate outlines from LBSR (162549). Pictures taken at 400x. Scale bar represents 10 mm.

The phytolith assemblages from PP5-6N, indistinct of the StratAgg or the sample type, were dominated by grass characteristic morphotypes and elongates without decorated margins, followed by dicot leaf phytoliths and irregular and indeterminate morphologies (Figs 5 and 6A–6C). Among grasses, GSSCs were identified in high frequencies, mostly from the rondel type (Fig 6D-6F) (S2 Table). Dicot leaves, spheroids, blocky morphotypes, epidermal appendages, elongate with decorated margins (mostly sinuate) and restio morphotypes (Fig 6G–6L) were identified in moderate frequencies. Articulated phytoliths from epidermal cells of dicot leaves showed a high variety of shape outlines (Fig 7). Spheroid echinates (palms), sedge phytolith morphotypes, fruits and wood/bark phytoliths from dicot plants, and stomata had the lowest frequencies among all the StratAggs at PP5-6N. Statistically, sedges, dicot leaves and wood/bark, spheroids, stomata, elongates without decorated margins, blockys and irregular/indeterminate morphologies showed significant differences among StratAggs (Fig 4 and S3 Table). It is important to note that the distribution of grass and restio phytoliths is not statistically different from random among StratAggs.

Specific details on the distribution of the phytolith assemblages (plant types and plant parts) among StratAggs from PP5-6N, based on the Kruskal-Wallis and the pairwise comparison (Dunn’s) tests, are given below:

- The highest frequencies of stomata and blocky morphotypes are found in the earliest occupation in the YBSR (~92–89 ka) (Dunn’s pairwise comparison test–S3 and S4 Tables).
- The LBSR (~89–75 ka) differs from the other StratAggs by having high frequencies of elongates with no decorated margins and by low frequencies of sedge, dicot leaf phytoliths and blocky morphologies (Dunn’s pairwise comparison test–S3 and S4 Tables). Although not statistically significant, spheroid echinate phytoliths were identified in three samples although in very low frequencies, and these were not observed in other StratAggs at PP5-6N (S3 Table).
- During the transition from MIS 5 to MIS 4, throughout the occupation of ALBS (~74ka), we did not observe statistically significant differences between the phytolith assemblages from ALBS and other StratAggs (S4 Table).
- Two sets of samples (at and inside the dripline) were collected from SADBS, dating to the beginning of MIS 4 (~73–68 ka). The phytolith assemblages varied depending on sample provenance, and this was statistically significant. At the dripline area irregular and indeterminate morphologies dominated and this constitutes the defining feature of this StratAgg (Dunn’s pairwise comparison test–S3 and S4 Tables). Inside the dripline area, dicot leaves dominated the phytolith assemblages constituting the defining feature (Dunn’s pairwise comparison test–S3 and S4 Tables).
- No significant differences were found between the SGS (~67–62 ka) and other StratAggs (Dunn’s pairwise comparison test–S3 and S4 Tables).
- None of the samples analysed from OBS2 (~65–60 ka) contained enough phytoliths for a reliable interpretation of the data.
- The BAS (~63–60 ka) differed from other StratAggs by the high presence of sedges (Dunn’s pairwise comparison test–S3 and S4 Tables).
- There were no significant differences between the phytolith assemblages of the BBCSR (MIS3, ~63–51 ka), and other (Dunn’s pairwise comparison test–S3 and S4 Tables).

## Discussion

### Phytolith provenience and preservation

In order to better interpret the phytolith record, and thus the plant presence in archaeological sites, there are two factors that need to be taken into account. The first is whether phytoliths represent plants that were introduced by humans, this being even more significant in the case of rock shelters than caves because of the higher chances of receiving water and daylight necessary for the growth of plants. The second is the phytolith state of preservation. Understanding phytolith dissolution is critical for avoiding bias when interpreting the data. In relation to the former, at PP5-6N, the identification of phytoliths in anthropogenic layers (dominated by combustion features) compared to the scarcity or absence of phytoliths in geogenic layers implies that plants were intentionally introduced to the site by human action and the phytoliths underwent little or no post-depositional movement.

The identification through FT-IR of wood-ash derived calcite attests to the past presence of firewood even in those samples with an absence or a low phytolith concentration, or the absence of wood/bark phytolith morphotypes. But this leads us to question whether this absence of phytoliths in some combustion features is due to post-depositional processes affecting phytolith preservation or to the use of plants that do not produce phytoliths. Cabanes and Shahack-Gross [115] showed that partial dissolution is responsible for the decrease of phytoliths in absolute quantities, and for the increase of morphotypes showing weathering and rugulate textures. This is because the stability of certain morphotypes differs even though mineral composition and solubility is the same. When sediments are chemically unstable, it should also be expected that the most fragile morphologies would be the first to disappear. Here we considered the association of the mineralogical composition of the sediments together with correlation measurements of the phytolith concentration against three taphonomic indicators, these being the total number of morphotypes identified, the percentage of weathered morphologies and the frequencies of fragile morphologies, in order to understand the state of preservation of phytoliths at PP5-6N (Table 2). No significant *p-values* were recorded for most of the measurements implying that there is inconclusive evidence about the significance of the association between the variables. We interpret these results as indicative of the low effect of taphonomic processes on the phytolith assemblages as a whole. These results lead us to consider the phytolith assemblages at PP5-6N as representative of the original plant phytolith input due to human intervention.

Samples from the ALBS and SADBS StratAggs contained very low phytolith concentrations and in most of the samples we did not identify the minimum number of phytoliths (>50) needed for a reliable interpretation of the plant component [95]. Aragonite was detected through FT-IR in most of the samples and this is one of the more unstable minerals that calcium carbonate forms. Its preservation in these StratAggs attests to the chemical stability of those sediments. The micromorphological study observed little chemical alteration of the sediments in the SADBS [32] and thus phytoliths should be relatively stable. Then, if dissolution processes did not take place here, the most plausible reason that explains the low phytolith concentration might relate to the type of fuel used rather than preservation and this will be a focus of interpretation below. Because aragonite was found in sediments and related to the presence of molluscs in the context of combustion features, there are two potential explanations as to why aragonite was preserved. The first explanation is the presence of low-intensity fires with a burning temperature below 400°C, since this is the minimum temperature at which aragonite starts transforming into calcite [123]. The second explanation accounts for a behavioural trait when, once the molluscs were consumed, the shells were discarded by disposing them in the now-extinguished hearths, favouring the preservation of the aragonite. But this remains an open question for future studies.

Finally, the absence of phytoliths in samples from the black layers at the uppermost StratAggs (OBS2, OBS1, SGS, DBCS and RBSR) together with the lack of calcite in sediments might be indicative of some postdepositional processes affecting mineral stability in the uppermost levels of PP5-6N. This is related to the location of these decalcified layers that are near to and partially below and outside the dripline [32]. Nonetheless, this association between absence of calcite and lack of phytoliths does not account for most of the samples from the BAS and BBCSR StratAggs, where most of the samples showed high phytolith concentration and low presence of calcite. Therefore, we suggest that phytolith preservation should be interpreted in combination with FT-IR spectra and micromorphology.

### Fire uses, foraging strategies and site occupation at PP5-6N

The presence of a large morphological variety of phytoliths is indicative of the diversity of plants used by the inhabitants of the shelter. This is particularly true with dicots, where the high variety of articulated phytoliths in hearths suggests a wide-ranging selection of trees and shrubs foraged by the inhabitants of the site and of a wide variety of plant fuel used during the oldest occupation at PP5-6N (YBSR and LBSR) during the last phases of MIS 5 (~92–75 ka).

We report for the first time the identification of restios in the South African archaeological record and this occurs ~90 ka at PP5-6N during the occupation of the YBSR StratAgg. The presence of restios is constant through the occupation of the site (~92 to 49 ka). The identification of restios in anthropogenic layers indicates the gathering and intentional introduction into the site of these plants by MSA hunter-gatherers inhabiting the south coast of South Africa. Restio plants are a diagnostic family of the Fynbos biome. Restios (together with other graminoids) have been, and still are, used traditionally in southern Africa for building, thatching and for the construction of brooms and sleeping mats ([124], and references therein). After the arrival of European settlers to the Cape, restios were mainly used for building and thatching, with *Thamnochortus insignis* Mast. being the most widely used species because of its long culms [124,125]. Evidence for the use of restios by San people during the contact period with European settlements is not well known. In contrast, Khoe-Khoe people, who were herders of both cattle and sheep, used mats made with different graminoids for housing and safekeeping the cattle, as these can be easily dismantled and loaded onto cattle for transport when wandering in search of richer pasture areas and water sources [126]. Such ethnographic evidence for the use of restios and other graminoids by past and current southern African inhabitants raises the question as to their use by MSA populations inhabiting the south coast of South Africa. At PP5-6N, restio phytoliths were found in contexts of combustion features so their use might be related to fire purposes and maybe with the aim of controlling the temperature. As for grasses, phytoliths from restios can also be found attached to the bark of certain trees [96]. Hence, their presence in hearth contexts can also be related to some extent as the result of wood contamination, although marginal as noted by the study of the modern plants from the area [96]. At Sibudu Cave, the presence of remains of burnt bedding was reported at ~77ka [127,128], and these were constructed mainly from sedges, other monocots and topped with aromatic, insecticidal and larvicidal dicot leaves (e.g. *Cryptocarya woodii*) [128]. Restios have similar characteristics to sedges in terms of shape, thickness and resistance, as well as in the traditional uses of the plants, which overlap considerably [124]. Taking into account the context, the ethnographic knowledge and other palaeoarchives, an additional explanation for the presence of restios in fire contexts might be that people inhabiting the south coast of South Africa during the Late Pleistocene used them as sleeping mats and these could have been placed by the fires so they could have been burned accidentally at some point, and mixed with the rest of the plant fuel, or intentionally as a way of disposing of old or infested bedding. Overall, the presence of restio phytoliths as far back as 92 ka at Pinnacle Point is indicative that South African MSA coastal foragers were aware of the distinctiveness of restio plants, which they might have been for fire purposes and possibly as mats.

The combustion features in the lowermost StratAgg deposits (mainly from the YBSR and LBSR) are generally intact, single hearths showing relatively small disturbance and trampling except in their periphery [32]. This is corroborated through phytoliths and FT-IR since wood-ash derived calcite was the main mineral component in only four out of the nine samples analysed from outside hearths, and only one contained high phytolith concentration, which indicates slight dispersion of ashes and burned remains. Few samples from below hearths showed high phytolith concentration and the presence of calcite, what must be explained by the reworking of the black layer and the soil occupation surface beneath the fire. This might be the reflection of a site maintenance behaviour by sweeping and/or dumping hearth deposits. However, some downward translocation of phytoliths from the black layer might also account for this.

Irregular morphotypes were mainly detected in white layers from the LBSR and these have been traditionally associated with the wood and bark of trees and shrubs [95,129]. Collura and Neumann [108] noticed the presence of these irregular morphologies mainly in bark. Our plant reference collection from the GCFR showed a rather higher presence of irregular phytoliths in the wood/bark of trees and shrubs than in other plant types [96]. Because they were mainly observed in samples from white layers, which also contained the highest concentration of wood-ash derived calcite, it is plausible that the majority of them came from wood used as fuel. Conversely, the calcite concentration in most of the samples collected from the black layers is minimal and irregular morphologies also detected but in much lesser amounts. Black layers from the LBSR were characterized by a high phytolith concentration with grasses being the dominant vegetal component, while woody phytoliths (both from the leaves and wood/bark) and restio phytoliths were also detected but in lesser frequencies. Previous results on modern reference material from other Mediterranean environments (Israel and Greece) suggested that the presence of grass phytoliths in archaeological hearths may be related, at least partially, to contamination since between 30 and 50% of the phytolith assemblage detected in the wood and bark of trees belonged to grasses as these are commonly attached to the bark of trees [95,129]. The results of our modern plant reference material also showed that the wood/bark and leaves of dicot plants contained grass phytoliths as a result of contamination, but in much lesser amounts (mean: 16,5%) [96]. This study also showed that, quantitatively, the grass phytolith presence as contamination in other plants but grasses are on average 90 times lower [see Table 2 in Esteban et al. [96]]. This suggests the grass phytolith contribution in the studied hearths has minimal contamination from wood, implying the intentional introduction of grasses into the shelter by past inhabitants.

The fire experiments conducted by Albert and Cabanes [130] showed that the micro-charcoal fraction concentrated the major presence of dicot leaf and monocot (mainly from the grass family) phytoliths and this was noteworthy when the fresh fuel was used. Furthermore, the authors also showed that when fresh fuel was used, less calcite was produced. Samples from black layers, mostly from the lowermost StratAggs (YBSR and LBSR) concur with these findings. Karkanas et al. [32] interpreted the internal microstratigraphy of combustion features from the LBSR as suggestive of the existence of several combustion events but for a relatively short period of time. In this scenario, we suggest that humans inhabiting Pinnacle Point during these short-term occupation events during MIS 5 may have built fast fires and used mainly grasses with some fresh wood from trees and/or shrubs. Because these combustion features are always associated with shellfish, it is plausible that we are faced with fires built for specific purposes, perhaps for shellfish cooking. This interpretation of the data regarding cooking shellfish on fire is further reinforced by an ethnographical study conducted on the Anbarra Australian aborigines; as it resembles one behaviour used to cook shellfish underneath hearths by building fast fires with small sticks and mostly grasses which only lasted for a few minutes [131].

Changes in the mineralogical component, and to a lesser extent the vegetal component, of the sediments through the occupation of PP5-6N were noteworthy between the lower StratAggs (YBSR, LBSR, and ALBS dated from ~92 to ~74 ka) and the SADBS (dating to ~73–68 ka). The micromorphological study also showed that the occupation character of PP5-6N changed from low-intensity activities, suggesting relatively small groups and/or relatively short occupations in the lowermost StratAggs, to larger groups and/or longer occupations in the upper SADBS StratAgg [32]. The phytolith assemblages at the dripline area of the SADBS differed from the rest of the StratAggs in the higher presence of irregular morphologies probably derived, as discussed above, from wood/bark [e.g. [95,96]]. The SADBS also differed from the rest of the StratAggs in the mineralogical composition of the sediments, with the SADBS presenting the highest concentration of wood-ash derived calcite among StratAggs. Albert and Cabanes [130] showed through experimental fires that the percentage of carbonates (ashes) was higher when fires were made with dry wood. This is because dry wood produces a more complete combustion of the plant material[130]. At Sibudu Cave in KwaZulu-Natal, higher presence of calcite in hearths was also related to high amounts of wood fuel [69,132]. Phytolith production in wood from South African trees and shrubs is generally low and thus wood may contribute little to the phytolith record in archaeological deposits located at the GCFR [96]. Thus, although phytoliths were not identified in high concentrations in the SADBS, the high concentration of wood-ash derived calcite indicates the extensive presence of fire events, and hearths being continuously fed, most probably with dry wood, and this is not seen at the older occupations of the site (YBSR and LBSR). This is indicative of a different exploitation strategy of plant resources and a different way of making fires compared to older YBSR and LBSR. These changes in the vegetal and mineralogical component are associated with the changes in human behaviour detected through the cultural material beginning around 74 ka ago, during the transition from the ALBS to SADBS, when a major change in stone tool raw material from predominantly quartzite to heat-treated silcrete occurs [4,21]. Despite the coastline being further away from Pinnacle Point (14 km on average) [133], the cultural material recovered showed that people were still exploiting the coast and transporting shellfish back to PP5-6N [32]. Micromorphology showed that the SADBS deposits consisted of overlapping combustion features with some *in situ* fine combustion features but in general trampling and raked out hearth remains dominated, indicating intense combustion activities and also intense human occupation [32]. This frequency of combustion features was also seen as indicative of frequent visits to the site, perhaps even on a seasonal basis [32]. In this scenario, it is likely that past inhabitants of Pinnacle Point during early MIS 4 (~73–70 ka) were collecting large amounts of dry wood intentionally, as it is easier to gather and transport, and intensively, to invest in fire production in order to create the proper conditions for the continuous practice of heating silcrete and the subsequent knapping process [4]. The phytolith analysis shows that people during MIS 4 at Pinnacle Point targeted dry wood at the same time as a major change in stone tool technology [4,21] and intensifying human occupation [32] on the heels of significant climate change [134]. This may reflect advanced planning and organization of multiple activities (e.g. collection of appropriate wood, collection of silcrete, heat treatment of silcrete) to support consistent heat treatment of silcrete to produce the bladelet and microlithic technology that appears at Pinnacle Point at this time.

### Comparison with Pinnacle Point 13B

**Fig 8:**
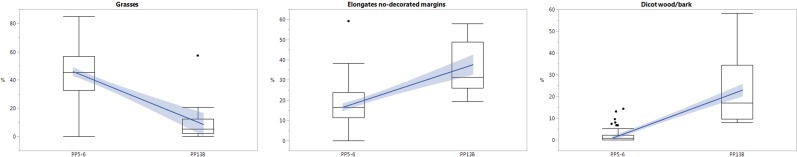
Comparison with PP13B. Box-Plots showing the distribution of grass, elongate without decoration margins, dicot leaf and wood/bark phytoliths among the different StratAggs from PP13B (Albert and Marean, 2012) (DB Sand 4c, Upper Roof Spall, Shelly Brown Sand and DB Sand 3 StratAggs) and PP5-6N (YBSR, LBSR, ALBS, SADBS and BBCSR StratAggs) sites. The median (mid-line), standard error ± (box), standard deviation (whiskers), outliers extended beyond the whiskers, and the trend line (or line of best fit) showing the confidence region for the fitted line are given for the four plant types.

There are notable differences in the vegetal component between PP13B [39] and PP5-6N, and this is suggestive of important changes in the patterns of plant foraging strategies by past hunter-gatherers inhabiting the south coast of South Africa through the Middle and Late Pleistocene. Our modern reference material from the study area [96,97] affords the opportunity to shed light on some puzzling results described by Albert and Marean [39] at PP13B. This site is a true cave and very well protected from the wind and rain with hearths located inside and within the dripline. The cave has a restricted floor space and therefore only one human band of people could fit comfortably inside. At PP13B the front and back of the cave have different preservation conditions [87], with better phytolith preservation at the back [39]. This results in a bias in the plant representation in the site because of the possible different plants used in different site areas [39]. Conversely, PP5-6N is a rock shelter and during the occupation of the site it would have been relatively exposed. It presents a much wider open area available for occupation so more people could conceivably inhabit it and/or spread themselves out more widely. Such differences might also be influenced by the mode of occupation of the two sites, shaping the strategies of the patterns of exploitation of the vegetal resources. At PP13B the vegetal component identified in some of the hearths from the back of the cave were mainly leaves from dicot plants. Noteworthy was that the identification of these dicot leaves took place during the oldest periods of occupation (middle MIS 6, DB Sand 4c StratAgg) [39]. Wood/bark phytoliths dominated the record in certain samples from the MIS 5c occupations of the Upper Roof Spall and DB Sand 3 [39] and these corresponded to periods of high-density occupation. Keeping in mind that the wood and bark of trees and shrubs from the central south coast of the GCFR produce very low numbers of phytoliths per gram of plant material [96], these high frequencies of wood/bark phytolith morphotypes is suggestive of the deliberate introduction of large quantities of wood in the cave and this is not observed at PP5-6N (Fig 8). Albert and Marean [39] interpreted the high abundance of dicot leaf phytoliths during DB Sand 4c (MIS 6) as the result of the production of fires with specific properties, including short-term activities or to a different use of activities such as cooking. Unfortunately, we have not been able to identify the taxonomic provenance of these dicot phytoliths in our plant reference material [96]. However, the high variability observed in the articulated phytoliths from dicot leaves found at both PP13B and PP5-6N might imply different plant provenance and thus a wide-ranging selection of trees and shrubs available and foraged by the inhabitants of Pinnacle Point through time.

With the exception of the samples from the Shelly Brown Sand (dating to MIS 5c), one common trait among the samples from PP13B deposits was the general low abundance of characteristic grass phytoliths [39]. Despite the bad preservation conditions of phytoliths in some of the areas of PP13B, Albert and Marean [39] interpreted the low grass phytolith abundance noted in well preserved samples as this family not being common in the surrounding areas of the cave. Based on the speleothem [135], macromammals [136] and strontium isotopes [30] records, we could expect the presence of extensive grasslands somewhere on the Palaeo-Agulhas Plain that was extensively exposed during MIS 6. If grasslands were present in the surrounding areas of PP13B during MIS 6, it is not detected in the phytolith record. Conversely, the high presence of dicot plants detected in the phytolith assemblage is representative of the presence of some sort of shrubby vegetation occurring in the vicinity areas of PP13B, and that this might have been the area preferred for plant foraging practices by the inhabitants of Pinnacle Point during MIS 6. Conversely, PP5-6N now shows that at Pinnacle Point people used grasses intensively in later periods. Thus, the results observed in both caves can indicate that the strategies of plant exploitation by people inhabiting Pinnacle Point changed overtime and possibly related to plant availability.

The high abundance of elongate morphologies with non-decorated margins–morphotypes that are considered to be originating from monocots–in most of the StratAggs of PP13B was ascribed to monocot plants, and related to grasses in those samples where characteristic grass phytoliths were identified [39]. At PP5-6N, although elongate morphologies with non-decorated margins were also identified in high frequencies, they are not as abundant as at PP13B (Fig 8). This is true even though phytoliths characteristic of restios and grasses are an important component of the phytolith assemblages at PP5-6N (see Fig 8) and these plants are also high producers of elongate morphologies [96]. Previous studies from Mediterranean plants [95,129] showed that elongate morphologies are common in monocots, and mostly in grasses. In the GCFR, Esteban et al. [96] analysed grasses, restios and geophytes all of them belonging to the monocot class. Geophytes lack diagnostic phytoliths although the leaves produced the highest numbers of elongate morphologies, and in particular those from the genera *Moraea* from the Iridaceae family, and this was statistically significant [96]. These results might point towards a monocot plant source of these elongate morphologies other than that of grasses and restios, and of its exploitation during the occupation of PP13B and mostly during the Upper Roof Spall and the Shelly Brown Sand StratAggs, dating to ~120–95 ka [39] (Fig 8). Could it be possible that this higher presence of elongate morphologies was related to the presence of geophytes? The GCFR is a rich flora of nutritious and forager-accessible USOs, most of which are cormous species belonging to the Iridaceae family [137]. It has been suggested that USOs, especially those belonging to the Iridaceae family [138], of the southern Cape could have supported the carbohydrate needs of a hunter-gatherer community over most of the year [138] and could also be used as a fallback food [26]. Despite the poor recording of the ethnobotany of the San and Khoe-Khoe people, it is known that geophytes, and specially *Moraea fugax*, were and still are, an important resource for food and water for San people from southern Africa [124,139,140]. Although records of plant species consumed is relatively well known [25,124,141–143], inferring plant-diet activities and harvesting strategies are very difficult as much of the hunter-gatherer tradition has been lost. Plant foods, and specifically the edible parts of tubers and corms, are usually fully consumed reducing the chances to find their remains in archaeological sites. We suggest that different species of geophytes were harvested in two different ways, by harvesting their entirety allowing the leaves to be passively transported and brought back into the cave, or by discarding the aerial parts during the processing in the field. We hypothesize that the first method of harvesting could have taken place at PP13B during MIS 5e, evinced by the high frequencies of elongate morphologies. Nonetheless, this hypothetical scenario is tentative and identifies the need for further comparative studies on highly diverse and endemic floras to increase knowledge and chances of plant identification in the fossil record, as well as the study of other archaeological sites from the southern coast of South Africa.

## Conclusions

This study explored the strategies of exploitation of vegetal resources by past hunter-gatherers inhabiting Pinnacle Point during the time span of the origins of modern humans. The main results of this study shed light on the fire fuel used, the mode of occupation of PP5-6N and changes in the strategies of plant exploitation by modern humans inhabiting the south coast of South Africa from ~160–49 ka.

We observed that plant phytoliths at PP5-6N were introduced into the site via anthropogenic input and that the phytolith assemblages can be considered representative of the original plant input. Even though partial dissolution might have affected the phytolith assemblages in some of the samples, this was not extensive enough to bias the interpretation of the whole data set.

This study reports the first evidence of the intentional gathering and introduction into living areas of restios by MSA hunter-gatherers inhabiting the south coast of South Africa during the Late Pleistocene. The restio phytoliths were identified in fire related contexts, and thus their presence may be related to fire purposes, or as sleeping mats that could have been accidentally burned and mixed with the rest of the plant fuel, or intentionally burned as a way of disposing of old or infested bedding. Nevertheless, the reason for the gathering and introduction of these plants into the shelter needs further research, as well as the study of phytoliths in other MSA archaeological sites for comparison.

Our study supports evidence for changes in plant foraging strategies that correlate with changes in lithic tool activities, site occupation intensity and climate change. We observed that during the last phases of MIS 5 the inhabitants of Pinnacle Point during these short-term occupation events used mainly grasses and woody plants to a lesser extent. Such a pattern is consistent with fast fires made mainly from grasses and some fresh branches from trees and/or shrubs collected from the vicinity of the site. Based on the phytolith and archaeological record and reinforced by ethnographical references we hypothesize that these combustion features were built to cook shellfish in hearths.

During MIS 4, together with the switch to technologies including regular use of heat treated silcrete and microlithic technology, we observe that people also changed plant foraging strategies by gathering large amounts of dry wood intentionally and intensively, and we infer this was done to improve combustion and to create the proper conditions for the regular practice of heating silcrete.

Further studies on contemporary archaeological South African sites are necessary to obtain a more complete picture on the uses of plants by modern human populations and their relation to the environment.

## Supporting information

S1 Table List of the one hundred eighty-three samples analysed from the PP5-6N sequence giving sample location and description, and the main phytolith, relative number of phytoliths per gram of sediment (/g sed) and FT-IR results, Arg, aragonite, Cal, calcite, Cl, clay (b = burned), (nb = not burned), (b? = probably burnt), Dah, dahllite, Nit, nitrate salts. Qtz, quartz.
(XLSX)

S2 Table List of phytolith morphotypes identified, their taxonomic affiliation and their frequencies in samples from the PP5-6N sequence, giving the stratigraphic location and sample information. (XLSX)

S3 Table Kruskal-Wallis test of rank sums of the distribution of the phytolith assemblages grouped by plant types and plant parts among the different StratAggs at PP5-6N. P-values in bold were detected as significant different among StratAggs.
(XLSX)

S4 Table Results of the Dunn's multiple comparisons test with Bonferroni adjustments for those plant types and plant types identified as significant through the Kruskal-Wallis test. (XLSX)
